# Ohio State Dental Society, December 8, 1898—Report of the Committee of Tri-State Meeting

**Published:** 1899-03

**Authors:** J. R. Callahan

**Affiliations:** Cincinnati


### PROCEEDINGS.

### Ohio State Dental Society, December 8, 1998—Report of the Committee of Tri-State Meeting.

BY J. R. CALLAHAN, D.D.S., CINCINNATI.

I think it was some time in the year 1893 that a union meeting of the State dental societies of Indiana, Michigan and Ohio began to be talked of. Possibly the first to mention or push the idea was Mr. C. S. Bigelow, of Toledo.

In 1893 Dr. G. H. Wilson, president of Ohio State Society, appointed what was known as the Tri-State Committee, consisting of Drs. L. L. Barber, J. Taft, G. H. Wilson.

This committee selected Dr. L. L. Barber as their representative on the Tri-State Executive Committee.

Through the influence of Dr. George Field, the first Tri-State meeting was held in Detroit. That meeting was a grand success and one that set the Ohio dentists to thinking of what they should do when the time should arrive to hold the meeting in the Buckeye State. During the administration of Dr. Henry Barnes, as president of the Ohio State Dental Society, he appointed the Tri-State Dental Meeting Committee as follows: Drs. L. L. Barber, L. E. Custer, A. F. Emminger, H. F. Harvey, and J. R. Callahan as chairman. This committee was called together at Put in-Bay Island, on the date of the meeting of the Northern Ohio Dental Society for 1897.

Plans for the future Tri-State meeting were discussed, but nothing definite was settled upon, except the fact that $1,000 would be necessary to see the meeting through in proper form. This being in the opinion of the committee a most important point.

The matter of subscription to this fund was brought up at one of the sessions of the Northern Ohio, when this energetic little society set the pace for the State by subscribing $100.

Soon after this meeting of the committee Indiana and Michigan suggested that each State elect some one member of the State committee to represent the State on an executive committee to have entire charge of carrying on the Tri-State meeting. With but one dissenting vote your committee elected their chairman to act as their representative on the executive committee. Thus the greater part of the responsibilities of organizing, providing funds and conducting this event of no small importance in the dental world was thrown upon the shoulders of one man. The chairman of your committee, realizing most keenly the responsibilities of the position, determined to allow no personal sacrifice to swerve him from carrying out the wishes of his State society.

The one desire of the Ohio State Dental Society being, as he understood it, that the Ohio dentists should prove to their guests that they were worthy the trust reposed in them.

In all undertakings of life it is wise to determine what are the fundamental principles upon which success depends. After due consideration it was decided that success depended, so far as the Ohio committee alone was concerned, upon some five or six points, or as might be termed fundamental principles, as follows:

1st. Entire harmony and unanimity of spirit throughout the State.

2d. A sufficient amount of cash, *in bank*, before a dollar was spent.

3d. Wise selection of place of meeting.

4th. Proper advertising.

5th. Special entertainment.

6th. Eternal, energetic push and hustle on the part of all concerned.

Now let us review briefly these six principles, as we have termed them.

First—Harmony and unanimity throughout the State. This was the least difficult of the six propositions to live up to, for an experience of some fifteen years has taught me that if the Ohio State Dental Society is once convinced of the righteousness of a cause, they will back it to the limit with action and purse. So I say it only required a little forethought, a trace of diplomacy and an honest effort, and to deal justly by all, to preserve that unity that brings strength. This is the greatest secret of our success.

Second—A sufficient amount of cash, *in bank*, before a dollar was spent. In how many States would it be possible to literally live up to this second proposition? It is with pride in my State that I tell you it was done in Ohio, in the season of '97 and '98. The subscriptions were as follows:

Charles I. Kelly, $10; J. R. Callahan, $10; O. N. Heise, $10; L. E. Custer, $10; L. P. Bethel, $10; C. H. Harroun, $10; L. L. Barber, $10; W. D. Snyder, $10; W. H. Hague, $10; E. G. Barnett, $5; A. F. Emminger, $10; W. H. Hersch, $10; A. E. McConkey, $10; J. A. Lupton, $10; C. R. Butler, $5; G. Molyneaux, $10: W. H. Todd, $10; F. A. Hunter, $10; J. F. Stephan, $10; S. B. Dewey, $10; H. F. Harvey, $10; G. S. Junkerman, $10; H. A. Smith, $10; J. Taft, $10; J. R. Owens, $10; W. A. Price, $10; Ruggles & Dennis, $10; H. C. Brown, $2; Cogswell & Co., $25; Cleveland Dental Co., $25; Ransom & Randolph, $25; S. A. Crocker & Co., $25; Northern Ohio Dental Society, $100; Cleveland Dental Society, $100; Charles Welch, $10; H. L. Ambler, $5; Henry Barnes, $10; Otto Arnold, $5; G. H. Wilson, $10; H. T. Smith, $5; H. Bartilson, $5; W. S. Locke, $5; W. T. McLean, $5; C. R. Converse, $5; P. S. Bollinger, $5; C. E. Tenney, $5; W. H. Tenney, $5; W. I. Jones, $10; Canton Dental Society, $27.50: Cincinnati Odontological Society, $100; A. T. Whitesides, $10; Akron Dental Society, $21; Toledo Dental Society, $77; Lee, Smith & Son, $25.

I should like to have had the individual subscriptions from the various local societies, but I did not think of it in time to procure them.

Total amount subscribed and paid - - $912.50

DISBURSEMENTS.

| | Voucher. | |
|---|---|---|
| Apollo Club transportation, hotel expenses from Cincinnati to Put-in-Bay - - - | 1. | $403.35 |

DISBURSEMENTS.

| | Voucher. | | |
|---|---|---|---|
| Hotel, Put-in-Bay - - | 2. | 156.75 | |
| Hotel punch bill - - - | 3. | 40.00 | |
| Committee expense bill - | 4. | 137.00 | —$737.10 |
| Balance on hand - - - - | | | $175.40 |

As this money was subscribed to a definite fund, it becomes your duty to turn it to some other fund by vote.

Third proposition—A wise selection of place for holding the meeting. This third proposition gave your representative no little trouble and anxiety. After many trials and vicissitudes the meeting was held at Hotel Victory, and carried through to a satisfactory close. But few complaints were placed on file. One party of juvenile excursionists became so attached to the boat on which they were traveling that they did not get off at Put-in-Bay—traveling on to Cleveland and return in order to get the value of their passage money. Good and hard they made a bluff protest to the captain, so that the extra fare might not be called for. The bluff worked. Then they tried the same game on your defenseless representative, who had to smile as if he believed the whole fake.

Fourth proposition—Advertisement. The attendance showed that the circular letters (being that portion of the advertisement detailed to your representative) did what they were intended to do.

The fifth point, viz—Special entertainment, was the most difficult question to dispose of. With the assistance of a few friends the program presented was decided upon. After the entertainment the smiles and hearty congratulations of many friends caused all recollections of trouble and excessively hard labor to vanish. On all hands people seemed to be well satisfied with what had been set before them. All this made your committeeman exceedingly happy.

The sixth and last proposition—Eternal push and hustle was well lived up to by all concerned. After the work was begun there was no let up. Printers, railroad people, steamboat people, hotel people, musical and theatrical people all over the country were followed up and annoyed till many of them I am sure were sorry they had begun with us.

The chairman of your Tri-State Committee desires to embrace this opportunity to thank the committee for their kind and prompt support whenever called upon. Making special mention of Dr. H. F. Harvey and to the society who supported every move made by the executive. And now after I have served you to the best of my ability for something like fifteen years, I request of you that I may be allowed to retire from executive committee work. There are others fully as competent, fully as willing.

DISCUSSION.

Dr. Hunter: Mr. President and Gentlemen—I will endeavor not to detain you more than a week or two, but in opening a discussion upon all scientific subjects it becomes necessary to begin at the beginning. In the year 1492 it is said Columbus discovered America, but not being present upon that occasion I cannot personally vouch for the fact, but in June, 1898, there was a meeting of the Tri-State Dental Society at Put-in-Bay, at which I was present, and can vouch, together with many others, for the eminent success of that entertainment. Dr. Callahan, as we all know, is the one man most to be blamed for the success of that meeting, which was through his hard work, work of which I had personal knowledge, knowing that he has at times neglected his own personal and private affairs to do work for the meeting of this society and in the engineering of that entertainment.

It was through him that the Apollinaris Club [laughter] of Cincinnati—thank you, I thought that was the name of the club—the Apollo Club. It originated in his brain to bring them to Put-in-Bay—to entertain us there. It is a musical organization of Cincinnati, of which Dr. C. is a member, but that should not be charged up against him. I am aware that some people think that the love of music is simply a slight form of hysteria, and they may be right, but the excellent program that the club rendered for us was certainly enjoyed by everybody present. The president of the society recently sent out communications for subscriptions, not stating the purpose of same, but it is now to be made apparent. I have been appointed a committee of one to make a presentation from this society as a slight token of our appreciation of the work done by Dr. Callahan upon that occasion.

We have here—John, there's your jug; take it! [Loud applause and laughter.]

A beautiful water and wine set was here presented to Dr. Callahan.

Dr. Callahan: Mr. President and Gentlemen—I know it is the custom on all such occasions to say you are taken completely by surprise, but I assure you it is a fact upon this occasion. I had no suspicion of anything of this sort. I know now why the president came to me and said he wanted $1, but I gave it to him. (Dr. Molyneaux here hands Dr. Callahan the dollar). I will take it and hold on to it just the same. I have his receipt for it; however I will keep the dollar. As to this beautiful present, of course I cannot express my feelings in regard to sentiment. I cannot make an extemporaneous speech. If I had known it I would have committed something to memory for an extemporaneous speech. It has always meant a great deal to me to know that my services were appreciated. I hope many of my friends in Cincinnati will drop in at any time and join with me in this, and those who live outside of Cincinnati I hope will do the same any time they are in the city and we will take a round together. Thank you. [Applause.]

Dr. H. A. Smith moved that the money left from Tri-State meeting be made a special fund to be used only by vote of the society. Carried.

Don't Forget General Wood.—The *Sample Bag* asks "Why did we beat Spain?" Because we are as strong as Sampson, we are as Schley as a fox, we are Miles long, we possess Merritt, we are Hobson's choice, what more Dewey want? . . . Only this—after the war is about over, the public in General Wood commit the care of the Cuban cities to a medical officer.

